# Mitochondria-Related Nuclear Gene Expression in the Nucleus Accumbens and Blood Mitochondrial Copy Number After Developmental Fentanyl Exposure in Adolescent Male and Female C57BL/6 Mice

**DOI:** 10.3389/fpsyt.2021.737389

**Published:** 2021-11-18

**Authors:** Cali A. Calarco, Megan E. Fox, Saskia Van Terheyden, Makeda D. Turner, Jason B. Alipio, Ramesh Chandra, Mary Kay Lobo

**Affiliations:** Department of Anatomy and Neurobiology, University of Maryland School of Medicine, Baltimore, MD, United States

**Keywords:** mitochondria, mitochondrial copy number, developmental drug exposure, fentanyl, nucleus accumbens, gene expression

## Abstract

The potency of the synthetic opioid fentanyl and its increased clinical availability has led to the rapid escalation of use in the general population, increased recreational exposure, and subsequently opioid-related overdoses. The wide-spread use of fentanyl has, consequently, increased the incidence of *in utero* exposure to the drug, but the long-term effects of this type of developmental exposure are not yet understood. Opioid use has also been linked to reduced mitochondrial copy number in blood in clinical populations, but the link between this peripheral biomarker and genetic or functional changes in reward-related brain circuitry is still unclear. Additionally, mitochondrial-related gene expression in reward-related brain regions has not been examined in the context of fentanyl exposure, despite the growing literature demonstrating drugs of abuse impact mitochondrial function, which subsequently impacts neuronal signaling. The current study uses exposure to fentanyl via dam access to fentanyl drinking water during gestation and lactation as a model for developmental drug exposure. This perinatal drug-exposure is sufficient to impact mitochondrial copy number in circulating blood leukocytes, as well as mitochondrial-related gene expression in the nucleus accumbens (NAc), a reward-related brain structure, in a sex-dependent manner in adolescent offspring. Specific NAc gene expression is correlated with both blood mitochondrial copy number and with anxiety related behaviors dependent on developmental exposure to fentanyl and sex. These data indicate that developmental fentanyl exposure impacts mitochondrial function in both the brain and body in ways that can impact neuronal signaling and may prime the brain for altered reward-related behavior in adolescence and later into adulthood.

## Introduction

Opioid use in the United States has dramatically increased in recent years, with death by opioid overdose reaching epidemic proportions (1, 2). Despite the high potential for opioid misuse and abuse, they remain some of the most effective treatments for pain management available. More recently, the synthetic opioid fentanyl, which is 50–100× more potent than morphine, has become both commonly prescribed and commonly added to illicit drugs increasing both use in the general population and opioid-related overdose deaths (1, 2). The rise in both use and misuse of opioids seen in the general population has also been observed among pregnant women, increasing both *in utero* exposure to opioids and increasing the occurrence of neonatal opioid withdrawal syndrome (NOWS) (3–6). Previous work in both humans and rodents has shown that developmental exposure to traditional opioids like morphine and heroin can lead to behavioral and developmental differences into adolescence and adulthood, including altered attention, stress responsivity, and learning and memory (7–10). Recently, studies examining the impact of developmental exposure to fentanyl have revealed changes in behavior and somatosensory processing into adolescence and adulthood (11, 12).

In addition to the many neurobiological changes in reward related processing, and cycles of negative affect that are associated with opioid use, escalation of use, and substance use disorders (13–18), opioid exposure is also associated with high degrees of oxidative stress and oxidative damage both centrally and peripherally (19–23). Patients with opioid use disorders show higher levels of oxidative and inflammatory markers in blood serum (24), and are more likely to show markers of metabolic syndrome, indicative of increased risk for mortality due to heart disease or diabetes (25). Multiple pre-clinical studies have shown metabolic disruptions and oxidative damage in brain tissue after morphine or heroin exposure (19, 26). Oxidative damage in the form of increased reactive oxygen species and decreased antioxidant enzyme activity caused by drug use can lead to mitochondrial dysfunction and neurotoxicity as well as other cellular damage (22, 26). Mitochondria specifically both absorb inflammatory and metabolic damage (27–29) and mediate brain function, neuroplasticity, and early life brain development (30–34).

Mitochondrial dynamics and changes therein due to stress, damage or altered energy requirements impact mitochondrial copy number, the ratio of mitochondrial DNA to nuclear DNA, which can be used as a proxy for mitochondrial function (35, 36). While brain tissue is not readily available from patient populations and does not allow for repeated sampling over the course of development, mitochondrial copy number in blood leukocytes is readily accessible in both clinical and pre-clinical samples. Indeed, understanding how peripheral blood-derived mitochondrial DNA copy number is associated with gene expression and mitochondrial function in other tissues is an active and important avenue of investigation (36). With respect to opioid use, mitochondrial copy number is reduced and markers of mitochondrial damage are increased in both human heroin users and rats exposed to chronic morphine (37). In the rats, mitochondrial copy number was also reduced in brain tissue, specifically the hippocampus (37). In cell culture, acute fentanyl and methadone, but not morphine, specifically negatively impact mitochondrial morphology and function (38, 39). It is currently unclear how the changes in mitochondria, accumulating mitochondrial damage, and other behavioral effects of opioid use are related. While mitochondrial function in reward-related brain areas, such as NAc, does regulate anxiety-like behaviors in rodent models (40–42), work on how altered mitochondria function contributes to increases in psychiatric symptoms and mood disorders in individuals with opioid use disorders or opioid exposure (43–45) is still needed.

The relationship between opioid use and mitochondrial function is still actively being explored and the current study sought to determine if developmental exposure to fentanyl causes long-lasting changes in peripheral and central markers of mitochondrial dynamics comparable to those observed after adult opioid use. Further, we sought to understand how peripheral markers of mitochondrial dynamics relate to mitochondrial gene expression in the reward-related brain regions critical for mediating opioid use, escalation of use, and opioid use disorder, specifically the nucleus accumbens (NAc) (46–48). Neuronal morphology and signaling changes in NAc have been shown to be critical for regulating both reward and anxiety- and depression-like behaviors (49, 50). Finally, we examined how both blood mitochondrial copy number and NAc gene expression correlated with behavioral measures of anxiety-like behavior and body weight. In this study we expand on the previously developed model of perinatal fentanyl exposure (11, 12) to explore the effects on blood mitochondrial copy number and expression of mitochondrial-related genes in NAc in adolescent mice. In this pre-clinical model, the perinatal period of mouse development consisting of gestation through weaning roughly corresponds to the full gestational period in humans, due to the developmental differences between species (51, 52). To our knowledge, this is the first study to examine mitochondrial copy number or NAc gene expression after developmental fentanyl exposure and subsequent forced abstinence.

## Methods

### Animals

All procedures were conducted in accordance with the Guide for the Care and Use of Laboratory Animals and approved by the Institutional Animal Care and Use Committees at the University of Maryland School of Medicine. Male and female C57BL/6J mice were bred to generate developmentally drug-exposed offspring in our facility. After verification of dam pregnancy by copulatory plug, sires were removed, and water was replaced with fentanyl-containing water or vehicle (see below). Vehicle controls received plain tap water. Water was monitored daily for consumption and replenished as necessary until litters were weaned at P21. After weaning, offspring were housed two to five per cage in single-sex groups, in a temperature- and humidity-controlled vivarium. Food and water were available *ad libitum*, and lights were maintained on a 12-h cycle.

### Drugs

At the time of pregnancy confirmation 10 μg/mL fentanyl citrate (Cayman Chemical; Cat# 22659) in tap water, or plain tap water (vehicle) was administered as the only source of available drinking water. This concentration has been reported previously for use in this developmental exposure model and was selected because mice will readily consume this dose, yet it does not cause motor deficits and is well-below LD50 of fentanyl in mice (11, 12). This dose has previously been shown to induce spontaneous withdrawal signs after weaning, but is not sufficient to disrupt maternal care behavior (11, 12).

### Behavior

After weaning at P21, offspring were left undisturbed until beginning behavioral testing. Each animal underwent an elevated plus maze (EPM) test and splash test, with 24 h separating each test. Twenty-four hours after the final behavioral test on day P35 body weight was measured and tissues were collected. The tissues used for analysis here were obtained from a subset of mice whose developmental exposure to fentanyl and behavioral testing results have been previously published by Alipio et al. (11). Tissue analysis was conducted on all 12 of the male water and male fentanyl mice, while a subset of the female mice were used: 17/22 female water and 17/31 female fentanyl mice. These mice include offspring from 7 different dams receiving water and 10 dams receiving fentanyl. Average litter size was 6 pups for both groups (Mean, SEM - Water: 6.14, 0.553; Fentanyl: 6.4, 0.476). Mice were habituated to the testing room before all behavioral procedures.

To measure anxiety-like behavior, mice were placed in the center of the EPM and were allowed to roam freely for 5 min, as described previously (11, 53, 54). Time spent in the open and closed arms of the maze in addition to the number of times the mouse entered one of the open arms were measured using computer tracking software (TopScan CleverSys, Reston, VA). Open/Closed ratios were calculated by dividing the time spent in the open arms by the time spent in the closed arms.

The splash test was used to measure affective state as has been described previously (11, 54). Mice were placed in an empty glass cylinder and their dorsal coat surface was sprayed three times with a 10% sucrose solution. Five min video recordings were experimenter scored by a blinded experimenter for time spent grooming.

### Tissue Collection

Twenty-four hours following the final behavioral assay, brains were removed, and trunk blood was collected. Blood was collected in a 1.5 mL microcentrifuge tube containing 10 μL EDTA (0.5 M, Invitrogen, Cat#15575) to reduce clotting, vortexed and stored at −80°C until further processing. Brains were place on ice, cut into 1 mm sections using a brain block (Braintree Scientific), and 14-gague punches surrounding the anterior commissure, encompassing both NAc core and shell, were collected (2 per animal). Tissue punches were stored at −80°C until further processing.

### DNA Extraction and Analysis

Trunk blood was thawed and homogenized to break up any clots. DNA was extracted from whole blood using a QiaAmp DNA Micro Kit (Qiagen, Germantown, MD; Cat# 56304) following manufacturer instructions. DNA quality and concentration were measured on a Nanodrop (Thermo Scientific), and DNA was diluted to 2 ng/μL for qPCR with PerfeCTa SYBR Green FastMix (Quantabio, Beverly, MA; Cat# 95072). To measure relative mitochondrial copy number, expression of the mitochondrial gene NADH dehydrogenase 1 (*mt-Nd1*) was compared to the nuclear gene glyceraldehyde 3-phosphate dehydrogenase (*Gapdh*) using the 2^−ΔΔCt^ method. Forward and reverse primer sets are as follows (F, R; 5′-3′): *Gapdh* AGGTCGGTGTGAACGGATTTG, TGTAGACCATGTAGTTGAGGTCA; *mt-Nd1* TACAACCATTTGCAGACGCC, TGTGAGTGATAGGGTAGGTGC. Data was further normalized within sex, such that male animals exposed to fentanyl were compared to male controls and female animals that received fentanyl were compared to female controls.

### RNA Extraction and Analysis

RNA was extracted from NAc tissue punches using Trizol (Invitrogen) and the MicroElute Total RNA Kit (Omega; Cat# R6831) with a DNase step (Qiagen, Germantown, MD; Cat# 79254). RNA quantity and concentration were measured on a Nanodrop (Thermo Scientific), and 400 ng of RNA was used to synthesize complementary DNA using a reverse transcriptase iScript complementary DNA synthesis kit (Bio-Rad, Hercules, CA; Cat# 1708891). Resulting cDNA was diluted to a concentration of 2 ng/μL, which was used to measure relative mRNA expression changes via quantitative PCR with PerfeCTa SYBR Green FastMix (Quantabio, Beverly, MA; Cat# 95072). Sixteen nuclear mitochondrial related genes were tested, and the primer sets are as follows (F, R; 5′-3′): *Cycs* TACATGCTACCACGGCTCTC, TGAGGTGACATGCCCCTATT; *Drp1* GGGCACTTAAATTGGGCTCC, TGTATTCTGTTGGCGTGGAAC; *Egr3* CCGGTGACCATGAGCAGTTT, TAATGGGCTACCGAGTCGCT; *Fis1* GGCTGTCTCCAAGTCCAAATC, GGAGAAAAGGGAAGGCGATG; *Mfn1* TATCGATGCCTTGCGGAGAT, GGCGAATCACAACACTTCCA; *Mfn2* GGAGACCAACAAGGACTGGA, TGCACAGTGACTTTCAACCG; *Nrf1* AGACCTCTGCTAGATTCACCG, CCTGGACTTCACAAGCACTC; *Nrf2* TCTACTGAAAAGGCGGCTCA, TTGCCATCTCTGGTTTGCTG; *Opa1* CAGCTCAGAAGACCTTGCCA, TCCTTCAACAAGCTGAGGCT; *Park2* GCACCTCAAGCAAGAATGAC, TACAGATGAGTGGGTCAGAGC; *Pgc1*α CGACCATGGTGTTGTTCTTG, ATGGCAGCGACTCCATACTC; *Pink1* GGGCTACTGTGTCCTGATGT, CTACTCCAGCTTGTCCCCTG; *Pol*γ ACTCCTGGAACAGTTGTGCT, CGTCCATCTACTCAGGACGG; *Tfam* TTTGTTGTGTGTGGGTGCTC, CGAAGGGCCATCCCTGTAT; *Tfb1m* TACGCCCTTGATAGAGCCCA, TCCTTCGAAACTGAAACGCA; *Tomm20* CTGTGCTCTGGGCACTTAAC, AGGGTGCACACAGGTCTAAT.

All biological samples were run in duplicate, and samples were excluded from analysis if duplicates were not within one CT value. Further, some samples did not yield sufficient RNA to run all genes tested, therefore, these samples were not run for all 16 genes. Quantification of mRNA changes was performed using the 2^−ΔΔCt^ method, using *Gapdh* and respective male and female control groups to normalize expression as described above.

### Statistics

All statistics were performed using GraphPad Prism version 9.1.2 for Windows (GraphPad Software, San Diego, California USA, www.graphpad.com). Behavioral data for the subset of mice used for tissue analysis were analyzed with two-way ANOVAs with Sidak *post-hoc* tests to compare within sex. Group data was analyzed for outliers using Grubb's-test, and outliers were removed from further analysis (no more than one per group). Relative DNA and RNA concentrations were compared within sex with unpaired *t*-tests when assumptions of equal variance were met and a Welch's corrected *t*-test when this assumption was violated. Data are presented as mean ± sem with individual data points overlaid. Simple linear regressions were used for correlations.

## Results

**Figure 1:**
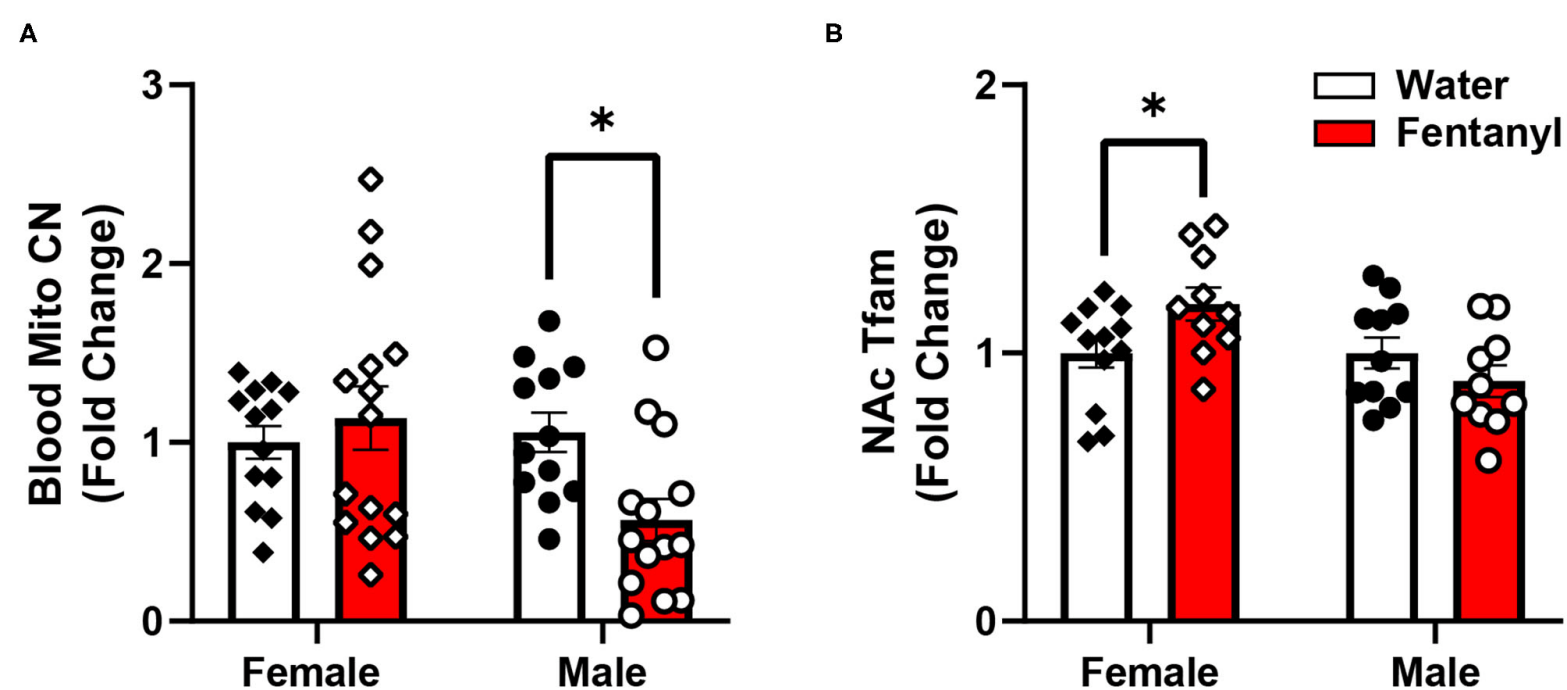
Mitochondrial copy number in blood and gene expression in NAc after developmental fentanyl exposure. **(A)** Developmental fentanyl decreases blood mitochondrial copy number in male, but not female adolescent mice. **(B)** Developmental fentanyl increases mRNA expression of Tfam in NAc in female, but not male mice. *p < 0.05.

To determine if developmental fentanyl exposure is sufficient to modulate peripheral mitochondria, we measured mitochondrial copy number in blood collected from P35 adolescent mice that had been exposed to fentanyl from conception through weaning. We did not observe an effect in female mice (Welch's corrected *t* = 0.677, df = 20.82, *p* > 0.05), however, blood mitochondrial copy number was significantly reduced in male mice that had received perinatal fentanyl as compared to control male mice (Figure 1A: *t* = 3.005, df = 24, *p* = 0.0061).

Opioid exposure and withdrawal in adulthood can cause both oxidative stress as well as gene expression changes in multiple brain regions (22, 25, 37, 39). Opioids are also highly addictive drugs with high abuse potential, and as such, readily engage reward-related brain circuitry during drug exposure (48, 55–58). Therefore, we examined the expression of multiple genes related to mitochondrial function in the NAc to determine if the peripheral changes in mitochondria are related to central changes in mitochondrial-related pathways in a reward-related brain region. These data are summarized in Table 1.

**Table 1:** Relative gene expression of nuclear mitochondrial related genes in NAc.

		**Control**			**Fentanyl**					
**Gene**	**Sex**	***n***	**Mean**	**95% CI**	***n***	**Mean**	**95% CI**	**Analysis**	***p*-value**	**Sig**
**Fission- and fusion-related genes**										
*Drp1*	Female	10	1.00	0.6707–1.329	8	0.8643	0.7428–0.9858	Welch's *t-*test	0.3798	n.s.
	Male	10	1.00	0.8393–1.161	8	0.8145	0.4416–1.187	*t-*test	0.2666	n.s.
*Fis1*	Female	12	1.00	0.820–1.180	11	1.067	0.8969–1.238	*t-*test	0.556	n.s.
	Male	11	1.00	0.9138–1.086	11	0.8957	0.7357–1.056	*t-*test	0.2157	n.s.
*Mfn1*	Female	12	1.00	0.8323–1.168	12	1.032	0.8772–1.187	*t-*test	0.7603	n.s.
	Male	11	1.00	0.9288–1.07	11	0.9784	0.8127–1.14	Welch's *t-*test	0.7938	n.s.
*Mfn2*	Female	12	1.00	0.9157–1.084	12	1.021	0.9245–1.118	*t-*test	0.7197	n.s.
	Male	12	1.00	0.9372–1.063	10	0.9929	0.8878–1.098	*t-*test	0.8943	n.s.
*Opa1*	Female	12	1.00	0.8864–1.114	9	1.022	0.9368–1.108	*t-*test	0.7443	n.s.
	Male	12	1.00	0.9033–1.097	11	1.076	0.9393–1.213	*t-*test	0.3194	n.s.
**Mitochondrial function, health, stress-resistance, mitophagy, and protein transport**										
*Cycs*	Female	9	1.00	0.7999–1.200	11	0.9385	0.7798–1.097	*t-*test	0.5868	n.s.
	Male	11	1.00	0.8917–1.108	9	1.735	0.7091–2.760	Welch's *t-*test	0.1383	n.s.
*Park2*	Female	10	1.00	0.8322–1.168	12	1.061	0.9342–1.189	*t-*test	0.5148	n.s.
	Male	11	1.00	0.9005–1.099	9	1.01	0.8131–1.207	*t-*test	0.916	n.s.
*Pink1*	Female	10	1.00	0.7716–1.228	12	0.7191	0.8210–1.287	*t-*test	0.7191	n.s.
	Male	11	1.00	0.8355–1.164	9	0.8873	0.6134–1.161	*t-*test	0.4136	n.s.
*Tomm20*	Female	10	1.00	0.8274–1.173	12	1.159	1.012–1.307	*t-*test	0.1313	n.s.
	Male	11	1.00	0.8941–1.106	9	0.925	0.6860–1.164	Welch's *t-*test	0.524	n.s.
**Nuclear transcription factors and transcriptional co-activators**										
*Egr3*	Female	12	1.00	0.7853–1.215	12	0.9545	0.8528–1.056	Welch's *t-*test	0.6794	n.s.
	Male	12	1.00	0.8669–1.133	11	1.125	0.8940–1.356	*t-*test	0.2994	n.s.
*Nrf1*	Female	11	1.00	0.8882–1.112	12	0.923	0.8101–1.036	*t-*test	0.2969	n.s.
	Male	11	1.00	0.9055–1.095	11	0.9437	0.7722–1.115	*t-*test	0.5289	n.s.
*Nrf2*	Female	12	1.00	0.7022–1.298	12	0.9898	0.7989–1.181	*t-*test	0.9502	n.s.
	Male	10	1.00	0.8929–1.107	9	1.234	0.4896–1.978	Welch's *t-*test	0.4934	n.s.
*Pgc1α*	Female	12	1.00	0.7647–1.235	10	0.9395	0.7330–1.146	*t-*test	0.6785	n.s.
	Male	10	1.00	0.8878–1.112	8	0.8442	0.4007–1.288	Welch's *t-*test	0.4451	n.s.
**Mitochondrial transcriptase and transcription factors**										
*Polγ*	Female	9	1.00	0.9216–1.078	10	1.059	0.8985–1.219	Welch's *t-*test	0.4693	n.s.
	Male	11	1.00	0.9416–1.058	7	0.99	0.5802–1.400	Welch's *t-*test	0.9549	n.s.
*Tfam*	**Female**	**12**	**1.00**	**0.8794–1.121**	**10**	**1.183**	**1.043–1.322**	***t-*** **test**	**0.0381**	*
	Male	11	1.00	0.8720–1.128	10	0.8953	0.7612–1.030	*t-*test	0.2207	n.s.
*Tfb1m*	Female	10	1.00	0.88490–1.151	9	0.946	0.8401–1.052	*t-*test	0.5231	n.s.
	Male	11	1.00	0.9189–1.081	6	1.142	0.6122–1.672	Welch's *t-*test	0.5254	n.s.

*Significant results in bold*.

* *p < 0.05, n.s. = not significant*.

We examined genes related broadly to multiple facets of mitochondrial function. Genes related to regulating the balance of mitochondrial fission and fusion to maintain mitochondrial number include the fission-related proteins dynamin-related protein 1 (Drp1) and mitochondrial fission 1 protein (Fis1), the fusion-related proteins mitofusin 1 and 2 (Mfn1, Mfn2), and OPA1 mitochondrial dynamin like GTPase (Opa1) (59). Gene expression for these fission and fusion-related proteins was not changed in NAc in either sex by developmental exposure to fentanyl. Genes involved in mitochondrial function, health, stress-resistance, mitophagy, and protein transport including cytochrome C (Cycs), parkin (Park2), PTEN induced kinase 1 (Pink1), and translocase of outer mitochondrial membrane 20 (Tomm20) (60) were also not changed in NAc in either sex after developmental fentanyl exposure.

Finally, we examined expression of genes related to transcription and transcriptional regulation of both nuclear and mitochondrial genome genes, broad regulators of overall patterns of gene expression (61, 62). Transcription factors and transcriptional co-activators that regulate nuclear genes related to mitochondrial function including Early Growth Response 3 (Egr3), nuclear respiratory factor 1 (Nrf1), nuclear factor, erythroid 2 like 2 (Nrf2), and peroxisome proliferator-activated receptor gamma coactivator 1-alpha (Pgc1α) were not changed in NAc in either sex after developmental fentanyl exposure. DNA Polymerase Subunit Gamma-1 (Polγ), a mitochondrial DNA polymerase which conducts mitochondrial DNA replication was not altered by developmental fentanyl, however, the mitochondrial transcription factor transcription factor A (Tfam) increased expression in female mice that had been developmentally exposed to fentanyl (Figure 1B). Tfam expression was not changed in male mice, nor were changes observed in either sex of transcription factor B1, mitochondrial (Tfb1). Notably, the change in expression of Tfam is double dissociated from the mitochondrial copy number finding in male mice.

**Figure 2:**
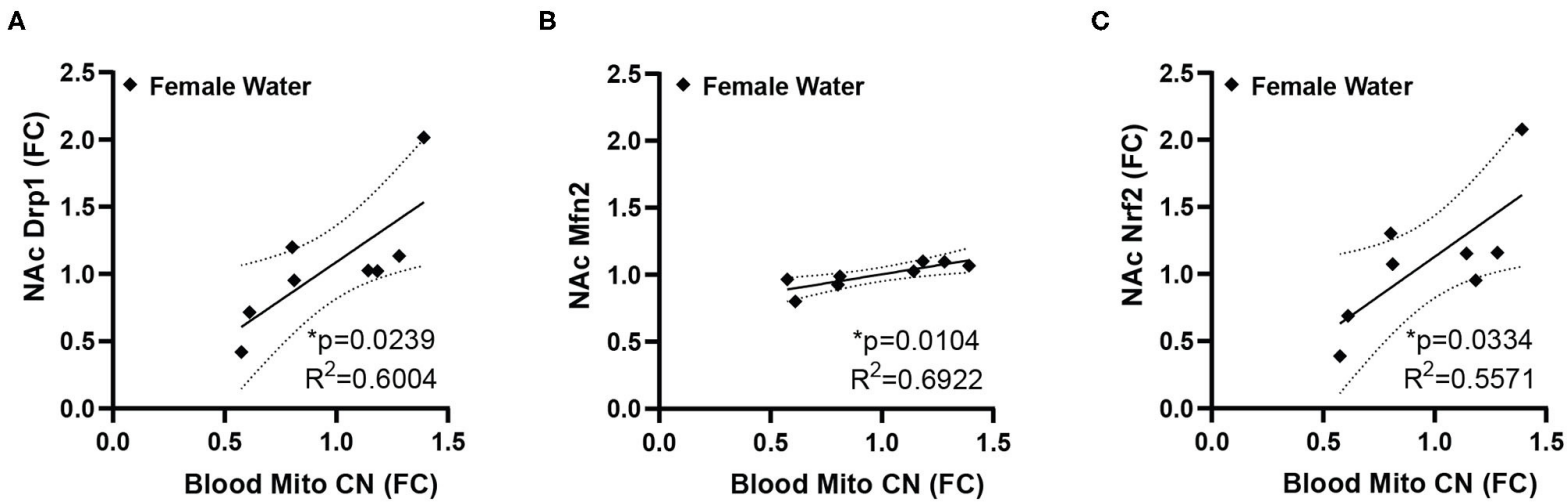
Blood mitochondrial copy number correlates with NAc gene expression in female mice. In female control mice blood mitochondrial copy number correlates with **(A)** NAc expression of Drp1 **(B)** Mfn2, and **(C)** Nrf2. Blood copy number and NAc gene expression are depicted as fold change. *p < 0.05.

While many of the genes examined did not show statistically significant differences, we noticed high degrees of variability in gene expression for many genes, therefore, we decided to explore if this variability in gene expression was related either to peripheral mitochondrial copy number, performance in the measured behavioral tests, or other factors that may relate to overall metabolic function, specifically body weight at the time of sacrifice. The behavioral data used here represents a subset of the previously published animals (11) used for the molecular analysis. In this subset of animals, male animals showed increased anxiety-like behavior in the EPM, indicated by a reduced ratio of time spent in the open arms of the maze over the time spent in the closed arms, but there was no effect in females (mean ratio, SEM - male control: 0.4081, 0.07036; male fentanyl: 0.1919, 0.02859; female control: 0.3661, 0.0441; female fentanyl: 0.3318, 0.0401) [main effect of drug *F*_(1, 54)_ = 7.419, *p* = 0.0087; *post-hoc* male adjusted *p* = 0.0082, female adjusted *p* > 0.05]. There was a main effect of sex in the splash test, with females spending more time grooming [*F*_(1, 54)_ = 10.48, *p* = 0.0021], however there was no effect of fentanyl or interaction (mean seconds, SEM - male control: 99.01, 5.497; male fentanyl: 81.83, 11.30; female control: 117.2, 4.954; female fentanyl: 112.8, 8.543). Finally, while males weighed more than females [*F*_(1, 54)_ = 5.622, *p* = 0.0213], we did not observe an effect of fentanyl on weight at this time point in this cohort (mean grams, SEM - male control: 18.21, 0.6542; male fentanyl: 18.82, 0.4767; female control: 17.2, 0.6829; female fentanyl: 17.11, 0.4271). We performed Pearson correlations on our copy number, gene expression and behavioral data and all correlations are described in Supplementary Table 1. Blood mitochondrial copy number showed a significant positive correlation with NAc Drp1, Mfn2, and Nrf1 in female control mice (Figure 2) but shows no relationship with NAc gene expression in female mice exposed to fentanyl or male mice of either condition. Specifically, there was no correlation between blood copy number with NAc Tfam expression in female mice, which was significantly increased in fentanyl-exposed mice. While developmental fentanyl does not change blood mitochondrial copy number in female adolescent mice, it does seem to disrupt the correlations with NAc gene expression seen in control mice, possibly indicating an uncoupling of peripheral and central mitochondrial function potentially unique to female mice, or a more complex relationship between NAc gene expression and blood mitochondria copy number in the context of developmental drug exposure.

**Figure 3:**
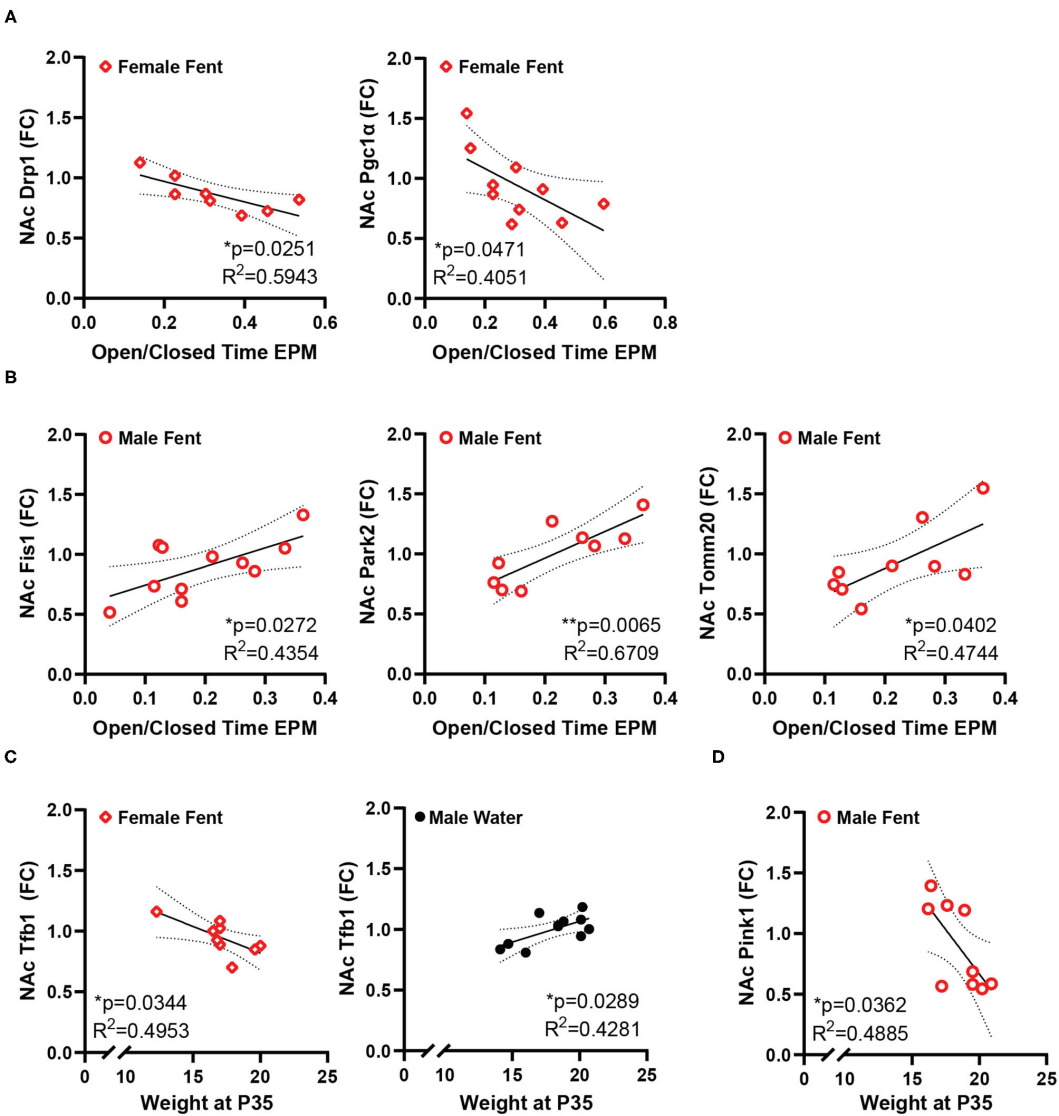
NAc mitochondrial gene expression correlates with anxiety-like behavior and weight. **(A)** In female mice developmentally exposed to fentanyl, NAc expression of Drp1 and Pgc1α correlate with behavior in the EPM, represented as the ratio of open/closed arm time. **(B)** In male mice developmentally exposed to fentanyl, NAc expression of Fis1, Park2, and Tomm20 correlate with behavior in the EPM, represented as the ratio of open/closed arm time. **(C)** NAc expression of Tfb1 positively correlates with weight at P35 in female mice developmentally exposed to fentanyl and negatively correlates with weight in male control mice. **(D)** In male mice developmentally exposed to fentanyl, NAc expression of Pink1 negatively correlates with weight at P35. Gene expression is depicted as fold change. *p < 0.05; **p < 0.01.

Conversely, the ratio of time spent in the open/closed arms of the elevated plus maze, an indicator of anxiety-like behavior, showed no correlation with gene expression in control animals, but is correlated with a number of genes in animals that had been exposed to fentanyl (Figure 3). In female mice EPM open/closed ratio negatively correlates with both Drp1 and Pgc1α expression in NAc (Figure 3A). In male animals, EPM open/closed ratio positively correlates with NAc expression of Fis1, Park2, and Tomm20 (Figure 3B). Time spent grooming in the splash test did not correlate with NAc gene expression for either sex under either drug exposure condition. Body weight positively correlated with NAc Tfb1 expression in male control mice, and negatively with Tfb1 in female mice exposed to fentanyl (Figure 3C). In male mice exposed to fentanyl body weight was negatively correlated with NAc expression of Pink1 (Figure 3D).

## Discussion

Opioid exposure is related to increased oxidative damage and mitochondrial damage in adulthood, and many negative impacts of chronic opioid use can be particularly long-lasting if the drug is encountered during development, leading to altered behavior and neurological function (9, 20, 24, 63). Opioid use and opioid use disorders are also associated with an increase in psychiatric mood symptoms as well as mood and anxiety disorders (43, 44). The current study examined how developmental exposure to the synthetic opioid fentanyl altered blood mitochondrial copy number, mitochondrial gene expression, and how these measures related to each other and anxiety-like behaviors in adolescent male and female mice. We showed that developmental exposure to fentanyl reduces blood mitochondrial copy number in male mice and increases NAc expression of Tfam mRNA in female mice. Additionally, mice exposed to fentanyl showed different patterns of correlation between blood mitochondrial copy number, anxiety-like behavior, weight, and NAc gene expression in a sex-dependent manner.

Mitochondria are particularly impacted by oxidative stress both as producers and scavengers of reactive oxygen species, and in turn are a critical mediator in downstream cellular processing and homeostatic changes in response to such oxidative stress. Mitochondrial DNA is particularly susceptible to oxidative damage compared to nuclear DNA, as it both lacks protective histone proteins and mitochondria have less robust DNA-repair machinery than nuclei (64). The mitochondrial genome is maintained in a highly dynamic equilibrium, existing in multiple copies per cell, with 1–10 copies per mitochondrion and multiple mitochondria per cell, depending on cell type (35, 65). Because the mitochondrial genome codes for most of the enzymatic subunits needed for oxidative phosphorylation, mitochondrial copy number can be used as an indicator of mitochondrial biogenesis (35, 66), and changes in mitochondrial copy number may contribute to oxidative stress, inflammation, and mitochondrial dysfunction (67, 68).

Mitochondrial dysfunction has been linked to disorders from diabetes, to cancer, and more recently to stress, psychiatric illnesses and substance use disorders (28, 69–74). Mitochondria copy number is increased in patients with bipolar disorder (75, 76), early childhood maltreatment or adversity (77), and increased in the prefrontal cortex and hippocampus of rats that had undergone cocaine self-administration, but is decreased in human heroin users and mice and rats exposed to chronic heroin (37). Our findings here mimic the decrease in copy number seen in male rodents, despite the developmental exposure and abstinence at the time of tissue collection used here. In the human patients, copy number did partially recover 3 months after initiation of heroin abstinence, although even after 6 months copy number had not fully recover to control levels (37). The clinical population represented female patients, although we did not observe changes in copy number in female mice. Further work will be needed to determine if opioids impact mitochondrial copy number comparably in men and women. Sex differences in substance use, substance use disorders, and successful abstinence have been readily observed for multiple used drugs, including opioids (78–82). A unique feature of the current study is the consistency of gestational fentanyl exposure *in utero* as our sample represents multiple litters consisting of both males and females. Fentanyl dose during the post-natal period was dependent on individual variance in pup milk or water consumption until weaning.

Although a growing number of studies are examining peripheral copy number, fewer studies are relating this measure to changes in other tissues, including the brain. In neurons, mitochondrial quality control and proper functioning impacts many aspects of cellular function related to signaling and circuit function (31). Specifically, in addition to providing the high levels of ATP necessary to maintain electro-chemical gradients, mitochondria buffer both intracellular calcium and reactive oxygen species, influence apoptosis, and have been shown to be critical for dendritic spine formation (31–33). Further, mitochondria and mitochondrial related genes in the NAc specifically have been shown to mediate behavioral responding for cocaine in a mouse model of substance use disorder (34, 83, 84), indicating mitochondrial function in NAc as a specific node for influencing the response to addictive drugs. Of the genes examined in NAc here, only Tfam showed increased expression. Interestingly, Tfam, because it binds to the mitochondrial DNA as a transcription factor, also has been shown to protect mitochondrial DNA from damage due to oxidative stress (61, 85, 86). Thus, it is possible there may be oxidative damage in NAc caused by developmental fentanyl exposure, and the increased expression of Tfam may be neuroprotective in female mice, consistent with their unchanged blood copy number. The sex-specific nature of this effect, and the lack of changes in other genes of interest may indicate tight regulation of mitochondrial function in NAc through development and during adolescence despite the drug exposure. Future studies should examine more direct measures of mitochondria in NAc, such as copy number. Heroin can reduce mitochondrial copy number in hippocampus indicating drug exposure can influence copy number in brain (37), but copy number can vary independently with brain region, and changes in one area may not predict changes in other connected brain regions (87). Importantly, differences in oxidative damage or gene expression may vary even within the NAc itself, as the NAc core, medial shell, and lateral shell all have previously described variance in regulating reward-related behaviors (88–92). The tissue used in this study included all subregions of the NAc, and future work will be needed to further dissect any unique subregion responses to developmental fentanyl. Mitochondrial morphology in NAc, regulated by many of the genes examined here, is also responsive to both drug exposure (34) and trait-anxiety measures possibly established during development (40). Fentanyl does impact mitochondrial morphology in neuronal-like NG108–15 cells (39), but this has not been demonstrated in any neuronal type *in vivo*. Further, the impact of developmental fentanyl exposure on mitochondria may be cell-type selective; beyond neurons, astrocyte function is altered by opioid exposure (93–95) and it is possible that changes in these glial cells or the immune-related microglia mimic changes seen in peripheral immune cells.

Our data indicate that blood mitochondrial copy number does vary systematically with NAc mitochondrial-related nuclear genes, specifically Drp1, Mfn1, and Nrf1, with higher copy number corresponding to higher gene expression. Drp1 regulates mitochondrial fission, promoting the formation of more mitochondria. In the brain, Drp1 is involved in new dendritic spine formation (33). While opioid exposure is linked to a decrease in dendritic spines, due to internalization of mu opioid receptors (96, 97), the correlation described here is in female mice specifically, which were resistant to both changes in blood copy and changes in EPM anxiety-like behavior, although fentanyl did disrupt the correlation with NAc gene expression. Mfn2 is usually considered a fusion-related protein (98), but it also plays critical role in mediating mitochondrial contact points with the endoplasmic reticulum independent of fusion and its expression in NAc has been linked to anxiety-like behaviors (40). Nrf1, as a nuclear transcription factor, regulates expression of other mitochondrial related genes including those related to mitochondrial respiratory function as well as genes involved in RNA metabolism, DNA damage repair, and ubiquitin-mediated protein degradation (99). While the role of mitochondrial related gene expression and function in NAc with respect to drug exposure is still a relatively new area of investigation, mitochondrial function within reward circuits has already been linked repeatedly to anxiety-like behaviors (41). Specifically, expression of mitochondrial related genes, mitochondria complex I and II function, and mitochondrial respiratory capacity impact both trait anxiety and expressions social dominance (40, 100, 101). Further, increases in ventral tegmental area dopamine input to NAc or D1-dopamine receptor agonism in NAc can increase both mitochondrial respiratory activity and facilitate social dominance expression in previously identified higher-anxiety rats (102). Both NRF1 and NRF2 regulate mitochondrial function in reward-related brain regions (103, 104), and global NRF2 knockdown is sufficient to decrease open arm time in rats in an EPM (104). NRF1 knockdown did not impact EPM behavior, but did alter expression of other mitochondrial-related proteins in the amygdala, hippocampus and prefrontal cortex (103). Since affect, mood, and anxiety are all impacted by drug use (57, 105–107), including opioid use (43, 44) and withdrawal (108), altered mitochondrial function may be a common underlying mechanism for trait anxiety or altered anxiety-like behavior after exposure to opioids and other drugs. Future work on both the genes of interest identified here and other mitochondrial processes is needed to fully understand their role in regulating behavior after drug exposure.

While these relationships with NAc gene expression exist in control animals, developmental fentanyl presented other relationships with gene expression, which negatively correlated with EPM behavior in female mice and positively in male mice. Both Drp1 and Pgc1α, the genes correlated in female mice, have both been shown in NAc to mediate enhanced behavioral responding to cocaine (34, 83), and here higher expression is related to less time spent in the open arms of the EPM. Conversely, Fis1, Park2, and Tomm20 all have functions in mitochondrial degradation pathways (109–112), potentially indicating higher degrees of mitochondrial damage or turn over in male mice after fentanyl which also have reduced blood copy number and significantly reduced open arm time in the EPM (11). Fentanyl also produced negative correlations between body weight and NAc expression of Pink1 in male mice and with Tfb1 in female mice. Pink1is protective against mitochondrial dysfunction (113), and as a mitochondrial transcription factor Tfb1 might have the same protective effects for mitochondrial DNA as Tfam. Future studies will be needed to further understand the relationship between weight, NAc gene expression, and opioid exposure. While some genes did correlate with behavior, Tfam did not, indicating that developmental fentanyl causes dissociable changes in the periphery and in NAc, and each of these tissues independently relate to behavior. Further, the sex differences seen here in both EPM behavior and in gene expression correlations are consistent with previous work showing important sex differences in NAc gene expression in the context of resilience to stress (114) and sex differences in the NAc proteome after exposure to nicotine, a commonly used drug (115).

It is important to note that in this study it is impossible to distinguish if the changes and relationships described here are due to the fentanyl exposure itself or due to the experience of going through withdrawal from the fentanyl after weaning. This regimen of fentanyl exposure is sufficient to induce spontaneous withdrawal signs (12), and it is unclear if the changes in behavior during adolescence are a prolonged result of withdrawal-related plasticity (58), or indicative of shifted baselines in stress reactivity caused by developmental insult (6, 9, 10). It is also possible, that the developmental timing of withdrawal (at weaning rather than at birth) may be significant. As mitochondria in the brain and body have been previously shown to modulate responses to both acute psychological stress (27), and mediate some of the developmental impacts on the brain of early-life stress (30), it is possible the effects seen here represent the mitochondrial response to opioid withdrawal (116–118). Future studies involving both continuous access to fentanyl and longer periods of abstinence into adulthood will be necessary to resolve this distinction and determine the persistence of these effects.

Taken together, these data indicate developmental fentanyl exposure has similar effects on offspring mitochondrial copy number as adult opioid use potentially including oxidative damage that disturbs mitochondrial function. Changes in the NAc are only one component of the reward circuits that may be impacted by this developmental opioid exposure and future work should examine the impacts on other brain regions, which may show stronger more significant relationships with blood mitochondrial copy number or behavior than those demonstrated here. The relationships with brain gene expression and behavior indicate coordinated responding throughout the body to the developmental insult of fentanyl exposure and future studies should further explore this relationship to better predict health and supplement treatment for infants with prenatal opioid exposure.

## Data Availability Statement

The raw data supporting the conclusions of this article will be made available by the authors, without undue reservation.

## Ethics Statement

The animal study was reviewed and approved by Institutional Animal Care and Use Committee at the University of Maryland School of Medicine.

## Author Contributions

ML, RC, CC, and MF were responsible for study concept and design. JA and MF designed the perinatal exposure paradigm. MF, JA, and MT managed breeding, fentanyl dosing, and behavioral testing. MF, JA, SVT, and CC collected tissues and performed experiments. CC performed data analysis. CC and ML drafted the manuscript. MF provided critical revision of the manuscript for content. All authors critically reviewed content and approved the final version for publication.

## Funding

This work was funded by NIH R01DA038613, NIH R01MH106500, T32DK098107, F32DA052966, and K99DA050575.

## Conflict of Interest

The authors declare that the research was conducted in the absence of any commercial or financial relationships that could be construed as a potential conflict of interest.

## Publisher's Note

All claims expressed in this article are solely those of the authors and do not necessarily represent those of their affiliated organizations, or those of the publisher, the editors and the reviewers. Any product that may be evaluated in this article, or claim that may be made by its manufacturer, is not guaranteed or endorsed by the publisher.

## Abbreviations

NAc: Nucleus Accumbens
EPM: elevated plus maze
Cycs: cytochrome C
Park2: parkin
Pink1: PTEN induced kinase 1
Tomm20: translocase of outer mitochondrial membrane 20
Drp1: dynamin-related protein 1
Fis1: mitochondrial fission 1 protein
Mfn1: mitofusin 1
Mfn2: mitofusin 2
Opa1: OPA1 mitochondrial dynamin like GTPase
Egr3: Early Growth Response 3
Nrf1: nuclear respiratory factor 1
Nrf2: erythroid 2 like 2
Pgc1α: peroxisome proliferator-activated receptor gamma coactivator 1-alpha
Polγ: DNA Polymerase Subunit Gamma-1
Tfam: mitochondrial transcription factor transcription factor A
Tfb1: Mitochondrial transcription factor B1.

## References

[1] Florence CS Zhou C Luo F Xu L. The economic burden of prescription opioid overdose, abuse, and dependence in the United States 2013. Med Care. (2016) 54:901–6. 10.1097/MLR.0000000000000625 27623005 PMC5975355

[2] Ryan SA. Calculating the real costs of the opioid crisis. Pediatrics. (2018) 141:18–21. 10.1542/peds.2017-4129 29507164

[3] Honein MA Boyle C Redfield RR. Public health surveillance of prenatal opioid exposure in mothers and infants. Pediatrics. (2019) 143:e20183801. 10.1542/peds.2018-3801 30655335 PMC6482836

[4] Haight SC Ko JY Tong VT Bohm MK Callaghan WM. Opioid use disorder documented at delivery hospitalization - United States, 1999-2014. Morb Mortal Wkly Rep. (2018) 67:845–9. 10.15585/mmwr.mm6731a1 30091969 PMC6089335

[5] Whiteman VE Salemi JL Mogos MF Cain MA Aliyu MH Salihu HM. Maternal opioid drug use during pregnancy and its impact on perinatal morbidity, mortality, and the costs of medical care in the United States. J Pregnancy. (2014). 2014:906723. 10.1155/2014/906723 25254116 PMC4164310

[6] Mactier H Hamilton R. Prenatal opioid exposure - increasing evidence of harm. Early Hum Dev. (2020) 150:105188. 10.1016/j.earlhumdev.2020.105188 32958331

[7] Šlamberová R. Drugs in pregnancy: the effects on mother and her progeny. Physiol Res. (2012) 61(Suppl. 1):932357. 10.33549/physiolres.932357 22827869

[8] Ross EJ Graham DL Money KM Stanwood GD. Developmental consequences of fetal exposure to drugs: what we know and what we still must learn. Neuropsychopharmacology. (2015) 40:61–87. 10.1038/npp.2014.147 24938210 PMC4262892

[9] Abu Y Roy S. Prenatal opioid exposure and vulnerability to future substance use disorders in offspring. Exp Neurol. (2021) 339:113621. 10.1016/j.expneurol.2021.113621 33516730 PMC8012222

[10] Franks AL Berry KJ DeFranco DB. Prenatal drug exposure and neurodevelopmental programming of glucocorticoid signalling. J Neuroendocrinol. (2020) 32:1–13. 10.1111/jne.12786 31469457 PMC6982551

[11] Alipio JB Brockett AT Fox ME Tennyson SS DeBettencourt CA El-Metwally D. Enduring consequences of perinatal fentanyl exposure in mice. Addict Biol. (2021) 26:e12895. 10.1111/adb.12895 32187805 PMC7897444

[12] Alipio JB Haga C Fox ME Arakawa K Balaji R Cramer N. Perinatal fentanyl exposure leads to long-lasting impairments in somatosensory circuit function and behavior. J Neurosci. (2021) 41:3400–17. 10.1523/JNEUROSCI.2470-20.2021 33853934 PMC8051687

[13] Koob GF. Neurobiological substrates for the dark side of compulsivity in addiction. Neuropharmacology. (2009) 56:18–31. 10.1016/j.neuropharm.2008.07.043 18725236 PMC2637927

[14] Volkow ND Koob GF McLellan AT. Neurobiologic advances from the brain disease model of addiction. N Engl J Med. (2016) 374:363–71. 10.1056/NEJMra1511480 26816013 PMC6135257

[15] Badiani A Blein D Epstein D Calu D Shaham Y. Opiate versus psychostimulant addiction: the differences do matter. Nat Rev Neurosci. (2008) 12:685–700. 10.1038/nrn3104 21971065 PMC3721140

[16] Shaham Y Erb S Stewart J. Stress-induced relapse to heroin and cocaine seeking in rats: a review. Brain Res Rev. (2000) 33:13–33. 10.1016/S0165-0173(00)00024-2 10967352

[17] Koob GF Volkow ND. Neurobiology of addiction: a neurocircuitry analysis. Lancet Psychiatry. (2016) 3:760–73. 10.1016/S2215-0366(16)00104-8 27475769 PMC6135092

[18] Crombag HS Bossert JM Koya E Shaham Y. Context-induced relapse to drug seeking: a review. Philos Trans R Soc B Biol Sci. (2008) 363:3233–43. 10.1098/rstb.2008.0090 18640922 PMC2607323

[19] Caspani G Sebok V Sultana N Swann JR Bailey A. Metabolic phenotyping of opioid and psychostimulant addiction: a novel approach for biomarker discovery and biochemical understanding of the disorder. Br J Pharmacol. (2021) 1–29. 10.1111/bph.15475 33817774

[20] Zahmatkesh M Kadkhodaee M Salarian A Seifi B Adeli S. Impact of opioids on oxidative status and related signaling pathways: an integrated view. J Opioid Manag. (2017) 13:241–51. 10.5055/jom.2017.0392 28953316

[21] Cunha-Oliveira T Rego A Oliveira C. Oxidative stress and drugs of abuse: an update. Mini Rev Org Chem. (2013) 10:321–34. 10.2174/1570193X113106660026

[22] Pavlek LR Dillard J Rogers LK. The role of oxidative stress in toxicities due to drugs of abuse. Curr Opin Toxicol. (2020) 20–21:29–35. 10.1016/j.cotox.2020.04.003

[23] Katz N Mazer NA. The impact of opioids on the endocrine system. Clin J Pain. (2009) 25:170–5. 10.1097/AJP.0b013e3181850df6 19333165

[24] Salarian A Kadkhodaee M Zahmatkesh M Seifi B Bakhshi E Akhondzadeh S. Opioid use disorder induces oxidative stress and inflammation: the attenuating effect of methadone maintenance treatment. Iran J Psychiatry. (2018) 13:46–54. 29892317 PMC5994232

[25] Molavi N Ghaderi A Banafshe HR. Short communication: examining metabolic profiles in opioid-dependent patient. Int J Med Toxicol Forensic Med. (2020) 10:1–6. 10.32598/ijmtfm.v10i3.28681

[26] Cunha-Oliveira T Rego AC Oliveira CR. Cellular and molecular mechanisms involved in the neurotoxicity of opioid and psychostimulant drugs. Brain Res Rev. (2008) 58:192–208. 10.1016/j.brainresrev.2008.03.002 18440072

[27] Picard M McManus MJ Gray JD Nasca C Moffat C Kopinski PK. Mitochondrial functions modulate neuroendocrine, metabolic, inflammatory, and transcriptional responses to acute psychological stress. Proc Natl Acad Sci. (2015) 112:E6614–23. 10.1073/pnas.1515733112 26627253 PMC4672794

[28] Picard M McEwen BS. Psychological stress and mitochondria: a systematic review. Psychosom Med. (2018) 80:141–53. 10.1097/PSY.0000000000000545 29389736 PMC5901654

[29] Picard M McEwen BS Epel ES Sandi C. An energetic view of stress: focus on mitochondria. Front Neuroendocrinol. (2018) 49:72–85. 10.1016/j.yfrne.2018.01.001 29339091 PMC5964020

[30] Hoffmann A Spengler D. The mitochondrion as potential interface in early-life stress brain programming. Front Behav Neurosci. (2018) 12:306. 10.3389/fnbeh.2018.00306 30574076 PMC6291450

[31] Rugarli EI Langer T. Mitochondrial quality control: a matter of life and death for neurons. EMBO J. (2012) 31:1336–49. 10.1038/emboj.2012.38 22354038 PMC3321185

[32] Li Z Okamoto K Hayashi Y Sheng M. The importance of dendritic mitochondria in the morphogenesis and plasticity of spines and synapses. Cell. (2004) 119:873–87. 10.1016/j.cell.2004.11.003 15607982

[33] Divakaruni SS Van Dyke AM Chandra R LeGates TA Contreras M Dharmasri PA. Long-term potentiation requires a rapid burst of dendritic mitochondrial fission during induction. Neuron. (2018) 100:1–16. 10.1016/j.neuron.2018.09.025 30318410 PMC6483400

[34] Chandra R Engeln M Schiefer C Patton MH Martin JA Werner CT. Drp1 mitochondrial fission in D1 neurons mediates behavioral and cellular plasticity during early cocaine abstinence. Neuron. (2017) 96:1327–41.e6. 10.1016/j.neuron.2017.11.037 29268097 PMC5747376

[35] Clay Montier LL Deng JJ Bai Y. Number matters: control of mammalian mitochondrial DNA copy number. J Genet Genomics. (2009) 36:125–31. 10.1016/S1673-8527(08)60099-5 19302968 PMC4706993

[36] Yang SY Castellani CA Longchamps RJ Pillalamarri VK O'Rourke B Guallar E. Blood-derived mitochondrial DNA copy number is associated with gene expression across multiple tissues and is predictive for incident neurodegenerative disease. Genome Res. (2021) 31:349–58. 10.1101/gr.269381.120 33441415 PMC7919448

[37] Feng YM Jia YF Su LY Wang D Lv L Xu L. Decreased mitochondrial DNA copy number in the hippocampus and peripheral blood during opiate addiction is mediated by autophagy and can be salvaged by melatonin. Autophagy. (2013) 9:1395–406. 10.4161/auto.25468 23800874

[38] Nylander E Grönbladh A Zelleroth S Diwakarla S Nyberg F Hallberg M. Growth hormone is protective against acute methadone-induced toxicity by modulating the NMDA receptor complex. Neuroscience. (2016) 339:538–47. 10.1016/j.neuroscience.2016.10.019 27746341

[39] Nylander E Zelleroth S Nyberg F Grönbladh A Hallberg M. The effects of morphine, methadone, and fentanyl on mitochondria: a live cell imaging study. Brain Res Bull. (2021) 171:126–34. 10.1016/j.brainresbull.2021.03.009 33741459

[40] Gebara E Zanoletti O Ghosal S Grosse J Schneider BL Knott G. Mitofusin-2 in the nucleus accumbens regulates anxiety and depression-like behaviors through mitochondrial and neuronal actions. Biol Psychiatry. (2021) 89:1033–44. 10.1016/j.biopsych.2020.12.003 33583561

[41] Filiou MD Sandi C. Anxiety and brain mitochondria: a bidirectional crosstalk. Trends Neurosci. (2019) 42:573–88. 10.1016/j.tins.2019.07.002 31362874

[42] Hollis F van der Kooij MA Zanoletti O Lozano L Cantó C Sandi C. Mitochondrial function in the brain links anxiety with social subordination. Proc Natl Acad Sci. (2015) 112:15486–91. 10.1073/pnas.1512653112 26621716 PMC4687564

[43] Rosoff DB Smith GD Lohoff FW. Prescription opioid use and risk for major depressive disorder and anxiety and stress-related disorders: a multivariable mendelian randomization analysis. JAMA Psychiatry. (2021) 78:151–60. 10.1001/jamapsychiatry.2020.3554 33175090 PMC7658804

[44] Gros DF Milanak ME Brady KT Back SE. Frequency and severity of comorbid mood and anxiety disorders in prescription opioid dependence. Am J Addict. (2013) 22:261–5. 10.1111/j.1521-0391.2012.12008.x 23617869 PMC6206504

[45] Martins SS Fenton MC Keyes KM Blanco C Zhu H Storr CL. Mood and anxiety disorders and their association with non-medical prescription opioid use and prescription opioid-use disorder: longitudinal evidence from the National Epidemiologic Study on Alcohol and Related Conditions. Psychol Med. (2012) 42:1261–72. 10.1017/S0033291711002145 21999943 PMC3513363

[46] Russo SJ Dietz DM Dumitriu D Malenka RC Nestler EJ. The addicted synapse: mechanisms of synaptic and structural plasticity in nucleus accumbens. Trends Neurosci. (2011) 33:267–76. 10.1016/j.tins.2010.02.002 20207024 PMC2891948

[47] Berridge KC Kringelbach ML. Pleasure systems in the brain. Neuron. (2015) 86:646–64. 10.1016/j.neuron.2015.02.018 25950633 PMC4425246

[48] Volkow ND Morales M. The brain on drugs: from reward to addiction. Cell. (2015) 162:712–25. 10.1016/j.cell.2015.07.046 26276628

[49] Fox ME Lobo MK. The molecular and cellular mechanisms of depression: a focus on reward circuitry. Mol Psychiatry. (2019) 24:1798–815. 10.1038/s41380-019-0415-3 30967681 PMC6785351

[50] Francis TC Lobo MK. Emerging role for nucleus accumbens medium spiny neuron subtypes in depression. Biol Psychiatry. (2017) 81:645–53. 10.1016/j.biopsych.2016.09.007 27871668 PMC5352537

[51] Clancy B Darlington RB Finlay BL. Translating developmental time across mammalian species. Neuroscience. (2001) 105:7–17. 10.1016/S0306-4522(01)00171-3 11483296

[52] Chen VS Morrison JP Southwell MF Foley JF Bolon B. Elmore SA. Histology atlas of the developing prenatal and postnatal mouse central nervous system, with emphasis on prenatal days E75 to E185. Toxicol Pathol. (2017) 45:705–44. 10.1177/0192623317728134 28891434 PMC5754028

[53] Pellow S Chopin P File SE Briley M. Validation of open: closed arm entries in an elevated plus-maze as a measure of anxiety in the rat. J Neurosci Methods. (1985) 14:149–67. 10.1016/0165-0270(85)90031-7 2864480

[54] Planchez B Surget A Belzung C. Animal models of major depression: drawbacks and challenges. J Neural Transm. (2019) 126:1383–408. 10.1007/s00702-019-02084-y 31584111 PMC6815270

[55] Luscher C Malenka RC. Drug-evoked synaptic plasticity in addiction: from molecular changes to circuit remodeling. Neuron. (2011) 69:650–63. 10.1016/j.neuron.2011.01.017 21338877 PMC4046255

[56] Nestler EJ Lüscher C. The molecular basis of drug addiction: linking epigenetic to synaptic and circuit mechanisms. Neuron. (2019) 102:48–59. 10.1016/j.neuron.2019.01.016 30946825 PMC6587180

[57] Kenny PJ Hoyer D Koob GF. Animal models of addiction and neuropsychiatric disorders and their role in drug discovery: honoring the legacy of Athina Markou. Biol Psychiatry. (2018) 83:940–6. 10.1016/j.biopsych.2018.02.009 29602521

[58] Thompson BL Oscar-Berman M Kaplan GB. Opioid-induced structural and functional plasticity of medium-spiny neurons in the nucleus accumbens. Neurosci Biobehav Rev. (2021) 120:417–30. 10.1016/j.neubiorev.2020.10.015 33152423 PMC7855607

[59] Yapa NMB Lisnyak V Reljic B Ryan MT. Mitochondrial dynamics in health and disease. FEBS Lett. (2021) 595:1184–204. 10.1002/1873-3468.14077 33742459

[60] Tilokani L Nagashima S Paupe V Prudent J. Mitochondrial dynamics: overview of molecular mechanisms. Essays Biochem. (2018) 62:341–60. 10.1042/EBC20170104 30030364 PMC6056715

[61] Kang D Hamasaki N. Mitochondrial transcription factor A in the maintenance of mitochondrial DNA: overview of its multiple roles. Ann N Y Acad Sci. (2005) 1042:101–8. 10.1196/annals.1338.010 15965051

[62] Ventura-Clapier R Garnier A Veksler V. Transcriptional control of mitochondrial biogenesis: the central role of PGC-1α. Cardiovasc Res. (2008) 79:208–17. 10.1093/cvr/cvn098 18430751

[63] Lee SJ Bora S Austin NC Westerman A Henderson JMT. Neurodevelopmental outcomes of children born to opioid-dependent mothers: a systematic review and meta-analysis. Acad Pediatr. (2020) 20:308–18. 10.1016/j.acap.2019.11.005 31734383

[64] Yakes FM Van Houten B. Mitochondrial DNA damage is more extensive and persists longer than nuclear DNA damage in human cells following oxidative stress. Proc Natl Acad Sci USA. (1997) 94:514–9. 10.1073/pnas.94.2.514 9012815 PMC19544

[65] Robin ED Wong R. Mitochondrial DNA molecules and virtual number of mitochondria per cell in mammalian cells. J Cell Physiol. (1988) 136:507–13. 10.1002/jcp.1041360316 3170646

[66] Phillips NR Sprouse ML Roby RK. Simultaneous quantification of mitochondrial DNA copy number and deletion ratio: a multiplex real-time PCR assay. Sci R. (2014) 4:3887. 10.1038/srep03887 24463429 PMC4894387

[67] Malik AN Czajka A. Is mitochondrial DNA content a potential biomarker of mitochondrial dysfunction? Mitochondrion. (2013) 13:481–92. 10.1016/j.mito.2012.10.011 23085537

[68] Zorzano A Liesa M Palaci M. Mitochondrial dynamics in mammalian health and disease. Physiol Rev. (2009) 89:799–845. 10.1152/physrev.00030.2008 19584314

[69] Ridout KK Khan M Ridout SJ. Adverse childhood experiences run deep: toxic early life stress, telomeres, and mitochondrial DNA copy number, the biological markers of cumulative stress. BioEssays. (2018) 40:1–10. 10.1002/bies.201800077 30067291

[70] Afrifa J Zhao T Yu J. Circulating mitochondria DNA, a non-invasive cancer diagnostic biomarker candidate. Mitochondrion. (2019) 47:238–43. 10.1016/j.mito.2018.12.003 30562607

[71] Wang X Sundquist K Rastkhani H Palmér K Memon AA Sundquist J. Association of mitochondrial DNA in peripheral blood with depression, anxiety and stress- and adjustment disorders in primary health care patients. Eur Neuropsychopharmacol. (2017) 27:751–8. 10.1016/j.euroneuro.2017.06.001 28647451

[72] Sadakierska-Chudy A Kotarska A Frankowska M Jastrzebska J Wydra K Miszkiel J. The alterations in mitochondrial DNA copy number and nuclear-encoded mitochondrial genes in rat brain structures after cocaine self-administration. Mol Neurobiol. (2017) 54:7460–70. 10.1007/s12035-016-0153-3 27819115 PMC5622911

[73] Silzer T Barber R Sun J Pathak G Johnson L O'Bryant S. Circulating mitochondrial DNA: New indices of type 2 diabetes-related cognitive impairment in Mexican Americans. PLoS ONE. (2019) 14:e0213527. 10.1371/journal.pone.0213527 30861027 PMC6414026

[74] Lee J-Y Lee D-C Im J-A Lee J-W. Mitochondrial dna copy number in peripheral blood is independently associated with visceral fat accumulation in healthy young adults. Int J Endocrinol. (2014) 2014:1–7. 10.1155/2014/586017 24707289 PMC3953665

[75] Wang D Li Z Liu W Zhou J Ma X Tang J. Differential mitochondrial DNA copy number in three mood states of bipolar disorder. BMC Psychiatry. (2018) 18:1–8. 10.1186/s12888-018-1717-8 29801445 PMC5970444

[76] Yamaki N Otsuka I Numata S Yanagi M Mouri K Okazaki S. Mitochondrial DNA copy number of peripheral blood in bipolar disorder: the present study and a meta-analysis. Psychiatry Res. (2018) 269:115–7. 10.1016/j.psychres.2018.08.014 30145290

[77] Tyrka AR Parade SH Price LH Kao HT Porton B Philip NS. Alterations of mitochondrial DNA copy number and telomere length with early adversity and psychopathology. Biol Psychiatry. (2016) 79:78–86. 10.1016/j.biopsych.2014.12.025 25749099 PMC4503518

[78] Bobzean SAM DeNobrega AK Perrotti LI. Sex differences in the neurobiology of drug addiction. Exp Neurol. (2014) 259:64–74. 10.1016/j.expneurol.2014.01.022 24508560

[79] Becker JB Koob GF. Sex differences in animal models: focus on addiction. Pharmacol Rev. (2016) 68:242–63. 10.1124/pr.115.011163 26772794 PMC4813426

[80] Fattore L Altea S Fratta W. Sex differences in drug addiction: a review of animal and human studies. Women's Heal. (2008) 4:51–65. 10.2217/17455057.4.1.51 19072451

[81] Lee CWS Ho IK. Sex differences in opioid analgesia and addiction: interactions among opioid receptors and estrogen receptors. Mol Pain. (2013) 9:1–10. 10.1186/1744-8069-9-45 24010861 PMC3844594

[82] Becker JB Chartoff E. Sex differences in neural mechanisms mediating reward and addiction. Neuropsychopharmacology. (2019) 44:166–83. 10.1038/s41386-018-0125-6 29946108 PMC6235836

[83] Chandra R Engeln M Francis TC Konkalmatt P Patel D Lobo MK. Role for peroxisome proliferator-activated receptor gamma coactivator-1α in nucleus accumbens neuron subtypes in cocaine action. Biol Psychiatry. (2017) 81:564–72. 10.1016/j.biopsych.2016.10.024 27939396 PMC5346327

[84] Cole S Chandra R Harris M Patel I Wang T Kim H. Cocaine-induced neuron subtype mitochondrial dynamics through Egr3 transcriptional regulation. Mol Brain. (2021) 14:101. 10.1186/s13041-021-00800-y 34187517 PMC8240292

[85] Xu S Zhong M Zhang L Wang Y Zhou Z Hao Y. Overexpression of Tfam protects mitochondria against β-amyloid-induced oxidative damage in SH-SY5Y cells. FEBS J. (2009) 276:3800–9. 10.1111/j.1742-4658.2009.07094.x 19496804

[86] Feng Q Shao M Han J Tang T Zhang Y Liu F. a potential oxidative stress biomarker used for monitoring environment pollutants in *Musca domestica*. Int J Biol Macromol. (2020) 155:524–34. 10.1016/j.ijbiomac.2020.03.208 32229201

[87] Fuke S Kubota-Sakashita M Kasahara T Shigeyoshi Y Kato T. Regional variation in mitochondrial DNA copy number in mouse brain. Biochim Biophys Acta - Bioenerg. (2011) 1807:270–4. 10.1016/j.bbabio.2010.11.016 21145305

[88] Laurent V Leung B Maidment N Balleine BW. μ- and δ-Opioid-related processes in the accumbens core and shell differentially mediate the influence of reward-guided and stimulus-guided decisions on choice. J Neurosci. (2012) 32:1875–83. 10.1523/JNEUROSCI.4688-11.2012 22302826 PMC3742880

[89] Peciña S Berridge KC. Dopamine or opioid stimulation of nucleus accumbens similarly amplify cue-triggered “wanting” for reward: entire core and medial shell mapped as substrates for PIT enhancement. Eur J Neurosci. (2013) 37:1529–40. 10.1111/ejn.12174 23495790 PMC4028374

[90] Di Ciano P Robbins TW Everitt BJ. Differential effects of nucleus accumbens core, shell, or dorsal striatal inactivations on the persistence, reacquisition, or reinstatement of responding for a drug-paired conditioned reinforcer. Neuropsychopharmacology. (2008) 33:1413–25. 10.1038/sj.npp.1301522 17712353

[91] Di Chiara G. Nucleus accumbens shell and core dopamine: differential role in behavior and addiction. Behav Brain Res. (2002) 137:75–114. 10.1016/S0166-4328(02)00286-3 12445717

[92] Castro DC Bruchas MR. A motivational and neuropeptidergic hub: anatomical and functional diversity within the nucleus accumbens shell. Neuron. (2019) 102:529–52. 10.1016/j.neuron.2019.03.003 31071288 PMC6528838

[93] Doke M Pendyala G Samikkannu T. Psychostimulants and opioids differentially influence the epigenetic modification of histone acetyltransferase and histone deacetylase in astrocytes. PLoS ONE. (2021) 16:e0252895. 10.1371/journal.pone.0252895 34115777 PMC8195369

[94] Lacagnina MJ Rivera PD Bilbo SD. Glial and neuroimmune mechanisms as critical modulators of drug use and abuse. Neuropsychopharmacology. (2017) 42:156–77. 10.1038/npp.2016.121 27402494 PMC5143481

[95] Kruyer A Chioma VC Kalivas PW. The opioid-addicted tetrapartite synapse. Biol Psychiatry. (2020) 87:34–43. 10.1016/j.biopsych.2019.05.025 31378302 PMC6898767

[96] Miller EC Zhang L Dummer BW Cariveau DR Loh H Law PY. Differential modulation of drug-induced structural and functional plasticity of dendritic spines. Mol Pharmacol. (2012) 82:333–43. 10.1124/mol.112.078162 22596350 PMC3400837

[97] Liao D Gringoriants OO Wang W Wiens K Loh HH Law PY. Distinct effects of individual opioids on the morphology of spines depend upon the internalization of mu opioid receptors. Mol Cell Neurosci. (2007) 35:456–69. 10.1016/j.mcn.2007.04.007 17513124 PMC1931568

[98] Adebayo M Singh S Singh AP Dasgupta S. Mitochondrial fusion and fission: the fine-tune balance for cellular homeostasis. FASEB J. (2021) 35:1–13. 10.1096/fj.202100067R 34048084 PMC8415099

[99] Satoh JI Kawana N Yamamoto Y. Pathway analysis of ChIP-seq-based NRF1 target genes suggests a logical hypothesis of their involvement in the pathogenesis of neurodegenerative diseases. Gene Regul Syst Bio. (2013) 2013:139–52. 10.4137/GRSB.S13204 24250222 PMC3825669

[100] Beyer F García-García I Heinrich M Schroeter ML Sacher J Luck T. Neuroanatomical correlates of food addiction symptoms and body mass index in the general population. Hum Brain Mapp. (2019) 40:2747–58. 10.1002/hbm.24557 30816616 PMC6865576

[101] Alonso-Caraballo Y Jorgensen ET Brown T Ferrario CR. Functional and structural plasticity contributing to obesity: roles for sex, diet, and individual susceptibility. Curr Opin Behav Sci. (2018) 23:160–70. 10.1016/j.cobeha.2018.06.014 31058203 PMC6497077

[102] Van Der Kooij MA Hollis F Lozano L Zalachoras I Abad S Zanoletti O. Diazepam actions in the VTA enhance social dominance and mitochondrial function in the nucleus accumbens by activation of dopamine D1 receptors. Mol Psychiatry. (2018) 23:569–78. 10.1038/mp.2017.135 28727688 PMC5822450

[103] Khalifeh S Oryan S Khodagholi F Digaleh H Shaerzadeh F Maghsoudi N. Complexity of compensatory effects in Nrf1 knockdown: linking undeveloped anxiety-like behavior to prevented mitochondrial dysfunction and oxidative stress. Cell Mol Neurobiol. (2016) 36:553–63. 10.1007/s10571-015-0236-0 26202310 PMC11482375

[104] Khalifeh S Oryan S Digaleh H Shaerzadeh F Khodagholi F Maghsoudi N. Involvement of Nrf2 in development of anxiety-like behavior by linking Bcl2 to oxidative phosphorylation: estimation in rat hippocampus, amygdala, and prefrontal cortex. J Mol Neurosci. (2015) 55:492–9. 10.1007/s12031-014-0370-z 25007950

[105] Haass-Koffler CL Bartlett SE. Stress and addiction: contribution of the corticotropin releasing factor (CRF) system in neuroplasticity. Front Mol Neurosci. (2012) 5:e00091. 10.3389/fnmol.2012.00091 22973190 PMC3434418

[106] Lüthi A Lüscher C. Pathological circuit function underlying addiction and anxiety disorders. Nat Neurosci. (2014) 17:1635–43. 10.1038/nn.3849 25402855

[107] Kutlu MG Parikh V Gould TJ. Nicotine addiction and psychiatric disorders. De Biasi M, editor. Nicotine Use in Mental Illness and Neurological Disorders. Amsterdam: Elsevier Inc. (2015). p. 171–208. 10.1016/bs.irn.2015.08.004 PMC5755398 26472530

[108] Koob GF. Neurobiology of opioid addiction: opponent process, hyperkatifeia, and negative reinforcement. Biol Psychiatry. (2020) 87:44–53. 10.1016/j.biopsych.2019.05.023 31400808

[109] Sprenger HG Langer T. The good and the bad of mitochondrial breakups. Trends Cell Biol. (2019) 29:888–900. 10.1016/j.tcb.2019.08.003 31495461

[110] Yu W Sun Y Guo S Lu B. The PINK1/Parkin pathway regulates mitochondrial dynamics and function in mammalian hippocampal and dopaminergic neurons. Hum Mol Genet. (2011) 20:3227–40. 10.1093/hmg/ddr235 21613270 PMC3140825

[111] Wai T Langer T. Mitochondrial dynamics and metabolic regulation. Trends Endocrinol Metab. (2016) 27:105–17. 10.1016/j.tem.2015.12.001 26754340

[112] Wang L Qi H Tang Y Shen H. Post-translational modifications of key machinery in the control of mitophagy. Trends Biochem Sci. (2020) 45:85–75. 10.1016/j.tibs.2019.08.002 31606339

[113] Narendra DP Youle RJ. Targeting mitochondrial dysfunction: role for PINK1 and parkin in mitochondrial quality control. Antioxidants Redox Signal. (2011) 14:1929–38. 10.1089/ars.2010.3799 21194381 PMC3078490

[114] Hodes GE Pfau ML Purushothaman I Ahn HF Golden SA Christoffel DJ. Sex differences in nucleus accumbens transcriptome profiles associated with susceptibility versus resilience to subchronic variable stress. J Neurosci. (2015) 35:16362–76. 10.1523/JNEUROSCI.1392-15.2015 26674863 PMC4679819

[115] Lee AM Mansuri MS Wilson RS Lam TT Nairn AC Picciotto MR. Sex differences in the ventral tegmental area and nucleus accumbens proteome at baseline and following nicotine exposure. Front Mol Neurosci. (2021) 14:657064. 10.3389/fnmol.2021.657064 34335180 PMC8317211

[116] Chartoff EH Carlezon WAJ. Drug withdrawal conceptualized as a stressor. Behav Pharmacol. (2014) 25:473–92. 10.1097/FBP.0000000000000080 25083570 PMC4321719

[117] Neugebauer NM Einstein EB Lopez MB McClure-Begley TD Mine3 ur YS Picciotto MR. Morphine dependence and withdrawal induced changes in cholinergic signaling. Pharmacol Biochem Behav. (2013) 109:77–83. 10.1016/j.pbb.2013.04.015 23651795 PMC3690589

[118] Kaufling J Aston-Jones G. Persistent adaptations in afferents to ventral tegmental dopamine neurons after opiate withdrawal. J Neurosci. (2015) 35:10290–303. 10.1523/JNEUROSCI.0715-15.2015 26180204 PMC4502267

