# Supplementary material for: Mitochondria-Related Nuclear Gene Expression in the Nucleus Accumbens and Blood Mitochondrial Copy Number After Developmental Fentanyl Exposure in Adolescent Male and Female C57BL/6 Mice
Source: Front Psychiatry. 2021 Nov 18;12:737389. doi: 10.3389/fpsyt.2021.737389 (PMC8637046; doi:10.3389/fpsyt.2021.737389)
Supplement: Supplementary file 1 [file Table_1.DOCX]

**Supplemental Table 1:** Correlation of blood copy number, NAc gene expression and anxiety-like behavior

|  |  | Blood CN | |  | EPM Open/Closed | | Splash Grooming | | Weight at P35 | |
| --- | --- | --- | --- | --- | --- | --- | --- | --- | --- | --- |
|  |  | Ctl | Fent |  | Ctl | Fent | Ctl | Fent | Ctl | Fent |
| EPM  Open / Closed | Female | -0.30 18 | 0.10  16 |  |  |  |  |  |  |  |
|  | Male | -0.04  11 | -0.45  10 |  |  |  |  |  |  |  |
| Splash Grooming | Female | 0.06  18 | 0.08  16 |  |  |  |  |  |  |  |
|  | Male | -0.17  9 | 0.12  10 |  |  |  |  |  |  |  |
| P35 Weight | Female | 0.11  18 | 0.28  16 |  |  |  |  |  |  |  |
|  | Male | 0.40  9 | 0.3  10 |  |  |  |  |  |  |  |
|  |  |  |  |  |  |  |  |  |  |  |
|  | Fission- and fusion-related genes | | | | | | | | | |
| *Drp1* | Female | **0.77***  **8** | -0.06  7 |  | -0.23 10 | **-0.77* 8** | 0.40  10 | -0.02 8 | 0.05  10 | 0.11  8 |
|  | Male | -0.65  7 | -0.41  7 |  | -0.5 10 | 0.39  8 | -0.16 10 | -0.52 8 | -0.17 10 | 0.31  8 |
| *Fis1* | Female | 0.30  8 | 0.06  9 |  | -0.19  12 | 0.36  11 | 0.47 12 | 0.28 11 | -0.28 12 | 0.15  11 |
|  | Male | -0.20  8 | -0.36  9 |  | -0.44 11 | **0.66* 11** | 0.34 11 | 0.11 11 | 0.39 11 | -0.14 11 |
| *Mfn1* | Female | 0.36  8 | -0.40  10 |  | -0.3  12 | -0.45 12 | 0.23  12 | -0.22 12 | -0.39 12 | 0.01 12 |
|  | Male | 0.07  8 | -0.63  9 |  | 0.17  11 | 0.53 11 | 0.19 11 | -0.13 11 | -0.30 11 | -0.51 11 |
| *Mfn2* | Female | **0.83***  **8** | -0.36  10 |  | -0.36 12 | -0.51 12 | 0.55 12 | -0.04 12 | -0.54 12 | -0.34 12 |
|  | Male | -0.10  8 | -0.17  8 |  | -0.15  12 | 0.28 10 | 0.33 12 | -0.01 10 | -0.17 12 | 0.15 10 |
| *Opa1* | Female | 0.42  8 | 0.01  7 |  | 0.03  12 | -0.41 9 | 0.01 12 | -0.22 9 | 0.36 12 | 0.44  9 |
|  | Male | -0.11  8 | 0.16  9 |  | 0.13  12 | 0.56  11 | -0.30 12 | -0.44 11 | -0.13 12 | -0.19 11 |
|  | Mitochondrial function, health, stress-resistance, mitophagy, and protein transport | | | | | | | | | |
| *Cycs* | Female | 0.10  6 | -0.56  9 |  | -0.05 9 | -0.19 11 | 0.62  9 | 0.43 11 | -0.23 9 | -0.59 11 |
|  | Male | -0.54 8 | 0.49  7 |  | -0.18 11 | 0.20 9 | -0.05 11 | -0.26 9 | 0.25  11 | 0.38 9 |
| *Park2* | Female | -0.10  7 | -0.10  10 |  | -0.16 10 | -0.03 12 | 0.39  10 | 0.39 12 | -0.37 10 | -0.45 12 |
|  | Male | 0.08  8 | -0.17  7 |  | -0.37 11 | **0.82** 9** | 0.17 11 | -0.64  9 | 0.33 11 | -0.13  9 |
| *Pink1* | Female | -0.22  7 | 0.02  10 |  | -0.34  10 | -0.45 12 | -0.10 10 | -0.04 12 | -0.25 10 | 0.06 12 |
|  | Male | 0.51  8 | -0.41  7 |  | 0.07  11 | 0.27  9 | 0.25  11 | -0.20 9 | 0.00 11 | **-0.70* 9** |
| *Tomm20* | Female | 0.15  7 | 0.34  10 |  | -0.13  9 | 0.30  12 | -0.23  9 | 0.30 12 | 0.33  9 | -0.09 12 |
|  | Male | -0.02  8 | 0.29  7 |  | 0.07  11 | **0.69* 9** | 0.07 11 | -0.25 9 | 0.16  11 | -0.17 9 |
|  | Nuclear transcription factors and transcriptional co-activators | | | | | | | | | |
| *Egr3* | Female | 0.37  8 | 0.30  10 |  | -0.10 12 | -0.36  12 | -0.13 12 | -0.14 12 | 0.26  12 | 0.18 12 |
|  | Male | 0.00  8 | 0.47  9 |  | -0.29 12 | 0.09 11 | 0.36  12 | -0.27 11 | -0.07 12 | 0.06 11 |
| *Nrf1* | Female | 0.14  8 | 0.14  10 |  | -0.6  11 | -0.37 12 | 0.07 11 | -0.24  12 | -0.52 11 | 0.23 12 |
|  | Male | -0.66  7 | -0.03  7 |  | -0.51  10 | 0.43  9 | -0.12 10 | -0.21 9 | 0.46 10 | 0.38  9 |
| *Nrf2* | Female | **0.75***  **8** | -0.48  10 |  | -0.20 12 | -0.07 12 | 0.45 12 | 0.03 12 | -0.07 12 | -0.15 12 |
|  | Male | -0.30  8 | -0.03  7 |  | -0.55 11 | 0.43 9 | 0.24 11 | -0.21 9 | 0.51  11 | 0.38 9 |
| *Pgc1α* | Female | 0.53  8 | -0.22  8 |  | -0.06 12 | **-0.64* 10** | 0.12 12 | 0.12 10 | -0.03 12 | -0.49 10 |
|  | Male | -0.68  8 | -0.14  6 |  | -0.01  10 | 0.28  8 | -0.42 10 | -0.27 8 | 0.07 10 | 0.49  8 |
|  | Mitochondrial transcriptase and transcription factors | | | | | | | | | |
| *Polγ* | Female | 0.41  6 | -0.48  8 |  | 0.07  9 | 0.10 10 | 0.66  9 | 0.05  10 | -0.40  9 | 0.07  10 |
|  | Male | -0.18  8 | 0.34  5 |  | -0.43 11 | -0.11 7 | 0.52 11 | -0.14 7 | 0.13 11 | 0.37  7 |
| *Tfam* | Female | 0.21  8 | -0.47  8 |  | -0.28 12 | -0.49 10 | 0.10  12 | -0.3 10 | -0.22 12 | -0.08  10 |
|  | Male | -0.05  8 | -0.21  8 |  | 0.13  11 | 0.58 10 | -0.11 11 | -0.18 10 | 0.14 11 | -0.38 10 |
| *Tfb1m* | Female | 0.64  7 | -0.31  7 |  | -0.14 10 | -0.53 9 | 0.41 10 | 0.10  9 | 0.13 10 | **-0.70* 9** |
|  | Male | -0.48  8 | 0.86  5 |  | -0.4  11 | 0.51  6 | 0.12 11 | 0.09  6 | **0.65* 11** | 0.44  6 |

Note: Cells contain the Pearson correlation coefficient and n. Significant results in bold; * p < 0.05, ** p < 0.01
